# Control System for Vertical Take-Off and Landing Vehicle’s Adaptive Landing Based on Multi-Sensor Data Fusion

**DOI:** 10.3390/s20164411

**Published:** 2020-08-07

**Authors:** Hongyan Tang, Dan Zhang, Zhongxue Gan

**Affiliations:** 1 Institute of AI and Robotics, Academy for Engineering & Technology, Fudan University, Shanghai 200433, China; hytang@fudan.edu.cn (H.T.); ganzhongxue@fudan.edu.cn (Z.G.); 2 Lassonde School of Engineering, York University, Toronto, ON M3J 1P3, Canada

**Keywords:** landing gear, adaptive landing, data fusion

## Abstract

Vertical take-off and landing unmanned aerial vehicles (VTOL UAV) are widely used in various fields because of their stable flight, easy operation, and low requirements for take-off and landing environments. To further expand the UAV’s take-off and landing environment to include a non-structural complex environment, this study developed a landing gear robot for VTOL vehicles. This article mainly introduces the adaptive landing control of the landing gear robot in an unstructured environment. Based on the depth camera (TOF camera), IMU, and optical flow sensor, the control system achieves multi-sensor data fusion and uses a robotic kinematical model to achieve adaptive landing. Finally, this study verifies the feasibility and effectiveness of adaptive landing through experiments.

## 1. Introduction

Compared with fixed-wing aircraft, vertical take-off and landing (VTOL) vehicles benefit from its multirotor power mode and have much fewer requirements for take-off and landing sites. VTOL vehicles are widely used in reconnaissance, search and rescue, logistics and other fields. The reduced requirements for the landing site lower the design requirements of the landing gear, but also limit the aircraft’s ability to take off and land on non-structural terrain.

To further expand the aircraft’s landing and landing environment, that is, to take off and land on complex unstructured terrain, more and more scholars have begun paying attention to the design of the adaptive landing gear of VTOL vehicles. The Mission Adaptive Rotor (MAR) project of the Defense Advanced Research Projects Agency (DARPA) organization was the first one to propose adaptive landing [1]. They adopted a legged mechanism to enable aircraft to adapt to different terrains. Subsequently, based on the difference in power output, two different types (active and passive) of adaptive landing gear were developed.

The active adaptive landing gear has developed into rigid-body landing gear and flexible-body landing gear. Rigid landing gear [2] mainly uses rigid connectors as the joints of the landing gear. The main representatives are the plane hinged robot landing gear of Edinburgh Napier University, Edinburgh, UK [3], the leg landing gear from Russia [4], the articulated leg landing gear of Kanazawa Institute of Technology in Japan [5], etc. All these landing gears apply legged mechanism to adjust touching points on the ground. The legged structure makes the robot’s modelling and controlling easier, but also make a challenge to driving motors on their hip joints. The driving motor keeps working all the time to keep the joints’ position, which may waste power energy. Flexible landing gear replaces joint motion by the deformation of flexible rods. The main representatives are the cable-driven landing gear of Georgia Institute of Technology [6], the avian landing gear [7] of UTHA University. The cable-driven landing gear uses spring dampers and cables to adjust its posture, and absorbs landing shock by the cable and spring damper. It is complicated but can be applied in heavy unmanned aerial vehicles (UAVs) and crewed aircrafts. The avian landing gear uses a soft gripper instead of a gild-body link, which can grip a rod and help vehicle standing on the rod. The passive landing gear is mainly powered by the weight of the robot and uses an under-actuated mechanism to achieve passive balance adjustment of the robot during the landing process, such as soft shock absorbers [8] of Imperial College London, a Four-bar linkage-based landing mechanism [9], and flexible landing gear [10] of China University of Petroleum.

Both positive and active landing gear can adjust posture by their mechanism. These mechanisms are the base hardware for adaptive landing. To complete the automatic landing, it also needs a control system to drive the mechanism. To realize the adaptive landing function, the aircraft needs to be based on mechanism configuration and the design of the control algorithm [11]. According to the different sensors used by robots, adaptive landing controllers can be divided into three categories. The first is the contact sensor, such as the tact switch [12,13], the pressure sensor [3,14], etc. These sensors are usually placed at the contact point of the landing gear on the ground and use a passive control method, which requires the relatively high real-time performance of the system. The second is visual sensors, which use three-dimensional visual scanning [15] to determine the terrain of the landing point, calculate the driving joint of the landing gear, and achieve adaptive landing. The third is the inertial measurement unit (IMU) [2,16]. IMU achieves adaptive landing by the different attitude control laws of the landing gear during landing. This control method requires higher design requirements for aircraft control algorithms.

On the other hand, computer vision is widely used in robotics. In UAV field, computer vision is applied in vision position [17,18] and visual recognition [19,20]. Vision position can calculate linear velocity with video data streams, and scan the 3D target with dual-camera. Vision recognition is applied in target and obstacle recognition. A depth camera is a novel vision sensor which can output both RGB image and depth image. The depth camera is a kind of low-cost 3D scanning approach comparing to 3D laser scanners, which is widely used in robot motion feedback [21], motion measurement [22], UAV obstacle avoidance [23], and other fields.

This article tries to apply the depth camera in VTOL UAV’s adaptive landing. Based on the hardware development foundation of the early landing gear robot of the laboratory team, this article combined with multi-sensor information, such as depth vision sensor, optical flow sensor, IMU, etc., data fusion, motion control of the landing gear robot to realize the aircraft adaptive control on complex unstructured terrain.

This article first introduces the main structure of the landing gear robot developed by authors. The next section introduces the mathematical basis of the control algorithm. Then the article proposes an adaptive landing control algorithm based on multi-sensor data fusion, and finally verifies the feasibility and effectiveness of the algorithm through experiments.

## 2. Adaptive Landing Gear Robot

**Figure 1:**
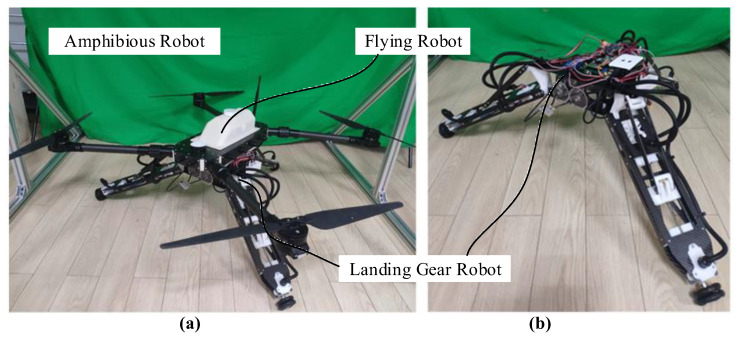
(**a**) The amphibious robot and (**b**) landing gear robot.

The landing gear robot analyzed in this paper is based on the tripod robot designed by authors. The amphibious robot (as shown in Figure 1a) can fly in the sky, dive underwater, and run on the ground. It is mainly composed of flying robots and landing gear robot. This article focuses on the control system in which landing gear robots land adaptively on unstructured terrain during the landing. This section introduces the components of the landing gear robot, including the mechanical structure, the power module, and the sensor module. This section lays the foundations for the following mathematical analysis and introduction of the control algorithm design.

### 2.1. Mechanical Structure

Figure 1b shows the landing gear robot studied in this article, which is mainly composed of a base and three limbs. The base is the main bearing part of the landing gear robot. The top of the base is connected and fixed with the flying robot through bolts. The base is mainly composed of three parts: Diving component, sensing component, and structural components. The structural components mainly use carbon fiberboard, and photosensitive resin, which are produced by cutting and 3D printing, and the parts are fastened and connected by screw nuts. The main function of the sensor component is to install the sensors required for the robot system, including the depth camera and optical flow sensor. The diving component is to achieve the robot’s diving function in the water, which is not the focus of this article.

**Figure 2:**
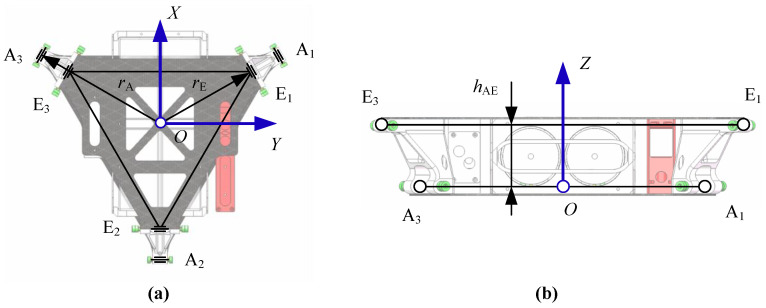
(**a**) Geometry structural and (**b**) dimensions of the base.

The top view of the base is as shown in Figure 2a. The geometric center of the base is defined as point *O*. The forward direction is the *X* axis direction of the base. The left direction is the *Y* axis. According to the right-hand rule, the direction of the vertical top surface is the *Z* axis direction. The center points of the six rotation axes connected with limbs are points A*_i_* and points E*_i_* (*i* = 1, 2, 3). Points A*_i_* and points E*_i_* are centrosymmetric around the *Z* axis. In the horizontal direction, the distance from Point A*_i_* and Point E*_i_* to the center point is *r*_A_ and *r*_E_, respectively.

**Figure 3:**
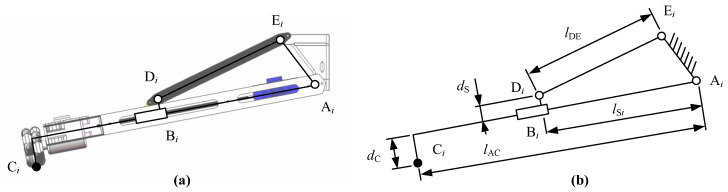
(**a**) Geometry structural and (**b**) dimensions of the limb.

The three limbs use the design of the slider link mechanism, shown in Figure 3. By driving the translation of slider B*_i_*, the swing rod A*_i_*C*_i_* is rotated, thereby controlling the height of the landing point C*_i_* of the landing gear robot. The schematic diagram of the mechanism is shown in Figure 3b. Point A*_i_* is the connecting point of the connecting rod D*_i_*E*_i_*, and the base and Point E*_i_* is the connecting point of the swing rod A*_i_*C*_i_* and the base. Point D*_i_* is the connecting point of the connecting rod and the slider. The length of the connecting rod is *l*_DE_. The length and eccentricity of the swing rod are *l*_AC_ and *d*_C_, respectively. The displacement and eccentricity of the slider are *l*_S*i*_ and *d*_S_, respectively.

The structure size parameters of the mechanism are shown in Table 1. The optimization of the structure size parameters of the structure is not the focus of this article and will not be discussed.

### 2.2. Power System

**Figure 4:**
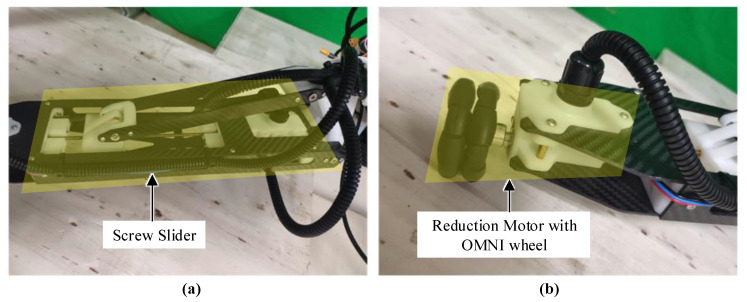
(**a**) Screw slider and (**b**) reduction motor with omnidirectional (OMNI) wheel.

The design and layout of the power system are related to the functional requirements of the landing gear robot. In this study, the landing gear robot mainly has two functions: Adaptive landing and omnidirectional motion. Therefore, this study designed two sets of power systems, as shown in Figure 4.

Figure 4a shows the screw slider assembly. The motor uses a DC reduction motor with an encoder. The structure of the leading screw allows the motor to keep the position of the slider (self-locking) when it stops rotating. Limit switches are installed at both ends of the leading screw to prevent the slider from locking beyond the stroke (locked-rotor). Figure 4b demonstrates an omnidirectional wheel assembly, which is mainly composed of a reduction motor and an omnidirectional wheel. The omnidirectional wheel uses an omnidirectional (OMNI) wheel with a diameter of 56 mm, and the reduction motor uses a DJI M2006 motor (made by SZ DJI Technology CO., Ltd., Shenzhen, China), which can realize the feedback of position, speed, and torque of the motor.

### 2.3. Sensor and Control System

**Figure 5:**
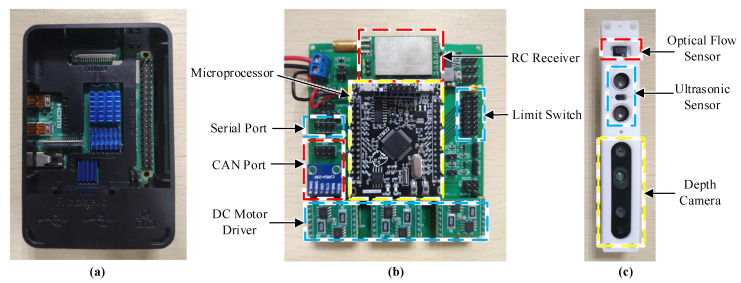
(**a**) Control computer, (**b**) driving board, (**c**) and sensor system.

The sensors of the robot mainly include depth cameras and optical flow sensors, show in Figure 5c. Depth cameras use Intel Realsense435i (made by Intel Corporation, Santa Clara, CA, USA), which can output image signals, depth signals, gyro signals, and acceleration signals. Depth cameras are mainly used for the detection of terrain during the descent of the robot. Optical flow sensor uses the lc302 optical flow (made by Youxiang Corporation, Changsha, China), which can detect the moving speed of the aircraft in the horizontal direction.

The control system of the robot is mainly composed of a control computer (show in Figure 5a) and a driving board (show in Figure 5b). The control computer uses the Raspberry Pi 4B (made by RS Components Ltd., Northants, UK) as the main carrier and is operating with the Ubuntu system. The control computer is mainly used for the analysis of image signals, the resolution of attitude signals, and the solution of robot kinematics (kinematical analysis). Once the driving joint variables are calculated, the control computer inputs it to the driving board to control the movement of the landing gear robot.

The driving board is mainly composed of a microprocessor, three DC motor drivers, and communication circuits. The microprocessor uses the STM32F405 chip (made by STMicroelectronics, Agrate, Catania, Italy) as the main processor for signal conversion and motor control. The DC motor drivers use the MOS chip for converting the processor’s electrical signal into the current required by the motor. The communication circuits, mainly integrated serial signal and controller area network (CAN) signal converter, are used to convert different signal modules.

## 3. Mathematical Analysis

This study is based on the mathematical modeling of the robot and the multi-sensor data fusion algorithm to achieve the adaptive landing function of the landing gear robot. The mathematical model of the robot is the mathematical foundation for realizing the robot motion control. The multi-sensor data fusion algorithm is the basis for realizing robot adaptive adjustment.

The basis for landing gear robot to achieve adaptive landing is terrain detection and analysis. Traditional mobile robots that perform three-dimensional reconstruction of environmental information can scan the terrain in a relatively stable state (variable movement oscillations are small). However, it is difficult for landing gear robots to keep stable because they are fixed to flying-submarine robots when they land. In order to maintain the attitude balance and fixed-point flight, it is difficult for the flying robot to ensure that the aircraft’s posture is in an ideal static state, which affects the detection of the terrain and the analysis of the landing point by the landing gear robot. Therefore, the landing gear robot’s judgment of the landing point needs to comprehensively consider the robot’s attitude angle, flight speed and other state information. This state information is collected by multiple sensors, and different sensor data have different characteristic information. In order to obtain complete, accurate, and real-time target state information, this study uses a complementary filter and Kalman filter to perform data fusion on multi-sensor signals.

### 3.1. Robot Mathematical Model

**Figure 6:**
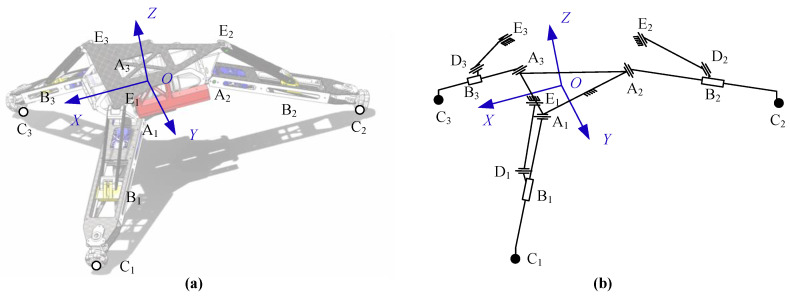
(**a**) The structural and (**b**) kinematic diagram of the landing gear robot.

The mathematical model of the robot is the basis for realizing robot motion control. In the previous section, the article introduces the robot mechanism and the simplified structure of the robot, as shown in Figure 6.

The main motion pair of the mechanism is the rotating pair and the moving pair. Our focus is on the correlation between the joint variable of the slider driving motor and the three landing points. The sketch map of the branch chain can clearly show the constraint relationship between the geometric structures of the mechanism:

(1) $$\begin{array}{c} \vec{E_{i}D_{i}}=\vec{E_{i}A_{i}}+\vec{A_{i}B_{i}}+\vec{B_{i}D_{i}}\\ \vec{O_{i}C_{i}}=\vec{O_{i}A_{i}}+\vec{A_{i}C_{i}} \end{array}\left(i=1,2,3\right)$$

The geometric constraints of the robot are converted into mathematical relations as follows:

(2) $$\begin{array}{l} \left|\vec{E_{i}D_{i}}\right|=norm(\vec{E_{i}A_{i}}+l_{Si}\cdot \vec{es_{i}}+d_{S}\cdot \vec{es_{i}}\times \vec{eA_{i}})=l_{DE}\\ \vec{O_{i}C_{i}}=\vec{O_{i}A_{i}}+l_{AC}\cdot \vec{es_{i}}+d_{C}\cdot \vec{eA_{i}}\times \vec{es_{i}} \end{array}$$

where $\vec{es_{i}}$ is the unit vector of the axis $\vec{A_{i}B_{i}}$; $\vec{eA_{i}}$ is the unit vector of the rotation axis $A_{i}$.

The forward kinematics and inverse kinematics of the robot can be solved by Equations (1) and (2).

In this study, we are mainly concerned with how to estimate the robot’s driving joint variables $l_{Si}$ through the height $h_{Ci}$ of the three contact points of the robot. Here we will introduce the solution process of inverse kinematics.

First, through the height of the contact point, the position and unit vector of the contact point *C_i_* can be obtained:

(3) $$\begin{array}{l} \vec{OC_{i}}=R_{AC,i}{\left(\begin{array}{ccc} d_{OCi} & d_{OCi} & -h_{Ci} \end{array}\right)}^{T}\\ \vec{es_{i}}=\frac{1}{\sqrt{2}}R_{AC,i}\cdot {\left(\begin{array}{ccc} \cos\left(\alpha_{i}\right) & \cos\left(\alpha_{i}\right) & \sin\left(\alpha_{i}\right) \end{array}\right)}^{T}\\ R_{AC,1}=\left(\begin{array}{ccc} 1/2 & 0 & 0\\ 0 & \sqrt{3}/2 & 0\\ 0 & 0 & 1 \end{array}\right),R_{AC,2}=\left(\begin{array}{ccc} -1 & 0 & 0\\ 0 & 0 & 0\\ 0 & 0 & 1 \end{array}\right),R_{AC,3}=\left(\begin{array}{ccc} 1/2 & 0 & 0\\ 0 & -\sqrt{3}/2 & 0\\ 0 & 0 & 1 \end{array}\right) \end{array}$$

where $d_{OCi}=\sqrt{l_{AC}^{2}-h_{Ci}^{2}}+r_{A}$ is the projected length of the vector $\vec{OC_{i}}$ on the horizontal plane; $\alpha_{i}$ is the angle between the axis $\vec{A_{i}C_{i}}$ and the horizontal plane; $R_{AC,i}$ is the coordinate conversion matrix.

Then, the obtained substitution $\vec{es_{i}}$ is substituted into Equations ((1) and (2)), and the driving rod length $l_{Si}$ can be obtained by solving the unary quadratic equation.

### 3.2. Complementary Filter

In this study, the robot’s attitude angle was calculated mainly by acquiring data from accelerometers and gyroscopes. The attitude angle can be obtained by integrating the angular velocity of the gyroscope. However, the gyro sensor has an integral drift, and the angle obtained by direct integration may contain errors. In order to eliminate the error of the sensor as much as possible, this study uses accelerometer data to correct the gyroscope data, and uses a complementary filter to solve the attitude angle.

The solution process of the complementary filter is mainly divided into five processes:

Normalize the acceleration data:

(4) $$a={\left(\begin{array}{ccc} a_{x} & a_{y} & a_{z} \end{array}\right)}^{T}=\frac{a_{z}}{norm(a_{z})}$$

where a is the normalized acceleration value, and $a_{z}$ is the value directly output by the accelerometer.

Convert the gravity vector $g_{Z}$ in the global coordinate system to body coordinates:

(5) $$g_{Z}=\left(\begin{array}{c} g_{Z,x}\\ g_{Z,y}\\ g_{Z,z} \end{array}\right)=\left(\begin{array}{c} 2\left(q_{1}\cdot q_{3}-q_{0}\cdot q_{2}\right)\\ 2\left(q_{0}\cdot q_{1}+q_{2}\cdot q_{3}\right)\\ q_{0}^{2}-q_{1}^{2}-q_{2}^{2}+q_{3}^{2} \end{array}\right)$$

Compensate the error e by doing a vector cross product in the body coordinate system:

(6) $$\begin{array}{l} e=\left(\begin{array}{c} e_{X}\\ e_{Y}\\ e_{Z} \end{array}\right)=a\times g_{Z}=\left(\begin{array}{c} a_{Y}\cdot g_{Z,z}-a_{Z}\cdot g_{Z,y}\\ a_{Z}\cdot g_{Z,x}-a_{X}\cdot g_{Z,z}\\ a_{X}\cdot g_{Z,y}-a_{Y}\cdot g_{Z,x} \end{array}\right)\\ e_{I}=\sum K_{I}\cdot e \end{array}$$

Calculate the proportional-integral (PI) of the error and compensate the angular velocity for compensate for the angular velocity:

(7) $$gyo_{k}={\left[\begin{array}{ccc} gyo_{x,k} & gyo_{y,k} & gyo_{z,k} \end{array}\right]}^{T}=gyo_{k-1}+K_{P}\cdot e+e_{I}$$

Update quaternions $q_{k}$:

(8) qk=Δt2(2Δt−gyox,k−gyoy,k−gyoz,kgyox,k2Δtgyoz,k−gyoy,kgyoy,k−gyoz,k2Δtgyox,kgyoz,kgyoy,k−gyox,k2Δt)⋅qk−1=Δt2Mq⋅qk−1

Convert quaternions to angles $\theta_{X}$ and $\theta_{Y}$:

(9) $$\begin{array}{l} \theta_{X}=\arctan\left(\frac{2q_{2}q_{2}+2q_{0}q_{1}}{1-2q_{1}^{2}-2q_{2}^{2}}\right)\\ \theta_{Y}=\arcsin\left(-2q_{1}q_{3}+2q_{0}q_{2}\right) \end{array}$$

### 3.3. Kalman Filter

The Kalman filter is a commonly used multi-information fusion method. It is an optimal estimation algorithm, which calculates the state variable of the system in a stepwise recursive manner to solve the minimum amount of estimated variance. In this study, the posture and speed of the robot are used as state variables, and the data of the gyroscope and optical flow sensor are used as the measured values.

To accurately determine the location of the robot’s landing point, this study will fuse the gyroscope and optical flow sensor data to calculate the robot’s position in the horizontal direction to further determine the robot’s landing point. When using a depth camera to scan the landing terrain, the camera is fixed at the center position of the robot. Therefore, the center point of the acquired depth image is used as the depth position corresponding to the center of the robot. Hence this study takes the relative displacement in the horizontal direction as one of the state variables of the filter. The state variables related to the relative displacement of the robot in the horizontal direction also include the angular velocity and linear velocity of the robot. The state variables of the filter can be expressed as:

(10) $$x_{k}=[\begin{array}{cccccc} \Delta P_{X,k} & \Delta P_{Y,k} & \omega_{X,k} & \omega_{Y,k} & v_{X,k} & v_{X,k} \end{array}]T$$

where $\Delta P_{i,k}(i=X,Y)$ represents the relative displacement of the robot at the k moment, $\omega_{i,k}$ represents the angular velocity of the robot at the k moment, and $v_{i,k}$ represents the linear velocity of the robot at the k moment.

When the robot is in the landing state, under ideal conditions, the robot’s horizontal speed and displacement approach zero, and the robot is in a relatively stable state. Therefore, in this study, the horizontal movement of the robot is approximated to a uniform speed for calculation, in other words, the state variable at the k moment can be predicted from the state variable at the previous moment:

(11) $${\hat{x}}_{k}=A*x_{k-1}=\left(\begin{array}{cccccc} 0 & 0 & 0 & 0 & \Delta t & 0\\ 0 & 0 & 0 & 0 & 0 & \Delta t\\ 0 & 0 & 1 & 0 & 0 & 0\\ 0 & 0 & 0 & 1 & 0 & 0\\ 0 & 0 & 0 & 0 & 1 & 0\\ 0 & 0 & 0 & 0 & 0 & 1 \end{array}\right)*x_{k-1}$$

where ${\hat{x}}_{k}$ are the predicted state variables and A is the state transition matrix.

According to the covariance at the k−1 moment, the predicted covariance ${\hat{P}}_{k}$ at the k moment can be calculated:

(12) $${\hat{P}}_{k}=A*P_{k}*A^{T}+Q_{k}$$

where $Q_{k}$ is the white noise that the system is interfered with by the outside world, and it is assumed to follow the standard normal distribution N(0,Q).

On the other hand, this study uses the data collected by the gyroscope and optical flow sensor to correct the current estimated state. The collected data and sensor errors are expressed as follows:

(13) $$\begin{array}{l} \mu_{z}=h_{k}={\left[\begin{array}{cccc} h\omega_{X} & h\omega_{Y} & hv_{X} & hv_{Y} \end{array}\right]}^{T}\\ \sigma_{z}^{2}=R_{k}={\left[\begin{array}{cccc} R\omega_{X} & R\omega_{Y} & Rv_{X} & Rv_{Y} \end{array}\right]}^{T} \end{array}$$

The relationship between sensor observations and state variables can be expressed by the following formula:

(14) $$\begin{array}{l} \mu_{H}=H_{k}*{\hat{x}}_{k}=\left(\begin{array}{cccccc} 0 & 0 & 1 & 0 & 0 & 0\\ 0 & 0 & 0 & 1 & 0 & 0\\ 0 & 0 & 0 & h_{C,k} & 1 & 0\\ 0 & 0 & h_{C,k} & 0 & 0 & 1 \end{array}\right)*{\hat{x}}_{k}\\ \sigma_{H}^{2}=H_{k}*{\hat{P}}_{k}*H_{k}^{T} \end{array}$$

where $H_{k}$ represents the conversion matrix between the state variable and the observed variable; $h_{C,k}$ represents the height of the robot at the current moment.

Both the measured value $\mu_{z}$ obtained and the predicted value $\mu_{H}$ obey the Gaussian distribution. Based on these two values, the optimal estimated value at the current moment can be calculated, which also satisfies the Gaussian distribution. According to the nature of the mean and variance of the Gaussian distribution, the mean and covariance of the best quality values can be respectively obtained as:

(15) $$\begin{array}{l} \mu_{k}=\mu_{H}+\frac{\sigma_{H}^{2}}{\sigma_{H}^{2}+\sigma_{z}^{2}}\left(\mu_{z}-\mu_{H}\right)=\mu_{H}+K\left(\mu_{z}-\mu_{H}\right)\\ \sigma_{k}^{2}=\sigma_{H}^{2}-\frac{\sigma_{H}^{2}}{\sigma_{H}^{2}+\sigma_{z}^{2}}\sigma_{H}^{2}=\sigma_{H}^{2}-K\sigma_{H}^{2} \end{array}$$

where K is the Kalman gain.

Sorting Equations (10)–(15), the update function of the Kalman filter can be obtained:

(16) $$\begin{array}{l} {\hat{x}}_{k}=A*x_{k-1}\\ {\hat{P}}_{k}=A*P_{k-1}*A^{T}+Q_{k}\\ K=H_{k}*{\hat{P}}_{k}*H_{k}^{T}{\left(H_{k}*{\hat{P}}_{k}*H_{k}^{T}+R\right)}^{-1}\\ xk={\hat{x}}_{k}+H_{k}^{-1}*K\left(h_{k}-H_{k}*{\hat{x}}_{k}\right)\\ P_{k}={\hat{P}}_{k}-H_{k}^{-1}*K*H_{k}*{\hat{P}}_{k} \end{array}$$

## 4. Control Algorithm Design

### 4.1. Adaptive Landing Process

In the traditional automatic landing process of a drone, the different stages of the drone landing are mainly determined based on the altitude information of the drone. The adaptive landing also needs to collect the environmental information of the landing site for comprehensive decision. In this study, adaptive landing is divided into two stages: The preparation stage and the descending stage.

During the preparation for the landing phase, the system predicts the landing posture of the landing gear robot by collecting terrain information. The control computer collects the sensor information, analyzes and calculates it, and then transmits the robot’s driving joint variables to the driving board through the serial port. Then the driving board drives the motors to change the robot’s attitude. During the descending phase, the flying robot controls the entire system to descend, and the control computer continues to analyze the terrain and make small adjustments to the robot’s landing attitude.

### 4.2. Algorithm Design

The key to realizing adaptive landing function is to convert the sensor information into the driving joint variables required by the robot. In this study, the sensor information mainly includes the depth image of the depth camera, the triaxle acceleration of the accelerometer, the triaxle angular velocity of the gyroscope, and the biaxial velocity of the optical flow module. The driving joint variables of the robot refer to the displacement of the driving sliders of the robot’s three limbs.

**Figure 7:**
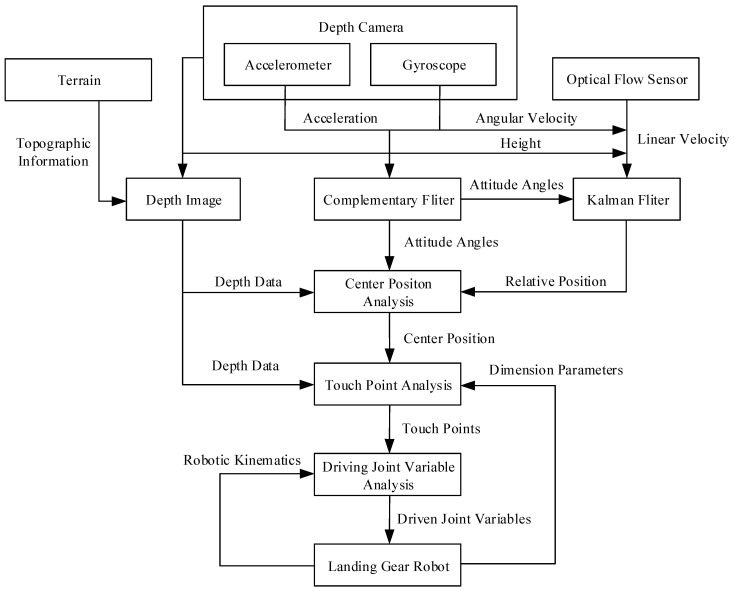
Flow chart of the adaptive landing control algorithm.

In this study, the information conversion is implemented in three steps: Center position analysis, contact position analysis, and drive variable analysis. The whole process is shown in Figure 7.

#### 4.2.1. Center Position Analysis

The center position refers to the position where the center of the robot landed on the ground, which is, the intended landing point. The determination of the location of the landing point is key to the robot’s adaptive terrain. In this study, we mainly discuss how to calculate the position of the center position in depth images.

**Figure 8:**
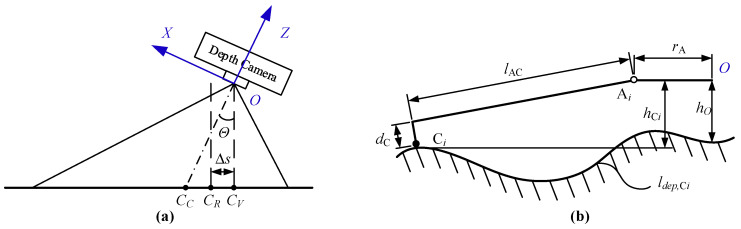
(**a**) Illustrations of center position analysis and (**b**) touch point analysis.

This study uses two steps to calculate the center position. First, calculate the vertical position of the robot center on the ground, based on the attitude angle of the robot, as shown in Figure 8a:

(17) $$C_{V}=C_{C}+K_{img}\cdot \Theta$$

where $C_{C}={\left(\begin{array}{cc} Pix_{X}/2 & Pix_{Y}/2 \end{array}\right)}^{T}$ is the pixel coordinates of the center position of the depth image; $K_{img}=\left(\begin{array}{cc} k_{img,X} & 0\\ 0 & k_{img,Y} \end{array}\right)$ is the ratio parameter between the depth image pixels and the angle; $\Theta ={\left(\begin{array}{cc} \theta_{X} & \theta_{Y} \end{array}\right)}^{T}$ is the attitude angle of the robot in the *X*-axis and *Y*-axis directions.

Then, according to the speed of the robot in the horizontal direction Δs, correct the center position $C_{R}$ of the robot at the next moment:

(18) $$\begin{array}{l} C_{R}=C_{V}+\Delta s=C_{V}+K_{img}\cdot \Theta_{s}\\ \Theta_{s}={\left(\begin{array}{cc} \arctan\left(\frac{\Delta P_{X,k}}{f_{dep}\left(C_{V}\right)}\right) & \arctan\left(\frac{\Delta P_{Y,k}}{f_{dep}\left(C_{V}\right)}\right) \end{array}\right)}^{T} \end{array}$$

where $f_{dep}$ is the depth surface function obtained by surface fitting according to the depth image at the k moment.

#### 4.2.2. Touch Point Analysis

Touch point refers to the contact point between the three limbs of the robot and the ground. Based on the center position $C_{R}$ of the robot, this study uses the geometric relationship of the robot limbs (as shown in Figure 8b) to calculate the robot’s touch point position.

First, calculate the depth curve $l_{dep,Ci}$ through the center of the robot:

(19) $$l_{dep,Ci}\left(t_{i}\right)=\left(\begin{array}{c} f_{len}\left(P_{img,i}\right)\\ f_{dep}(P_{img,i}) \end{array}\right),P_{img,i}=R_{AC,i}\cdot t_{i}+C_{R}$$

where $P_{img,i}$ is the coordinate position of the *i*-th limb in the depth image, $t_{i}$ is the parameter variable; $R_{AC,i}\left(R_{AC,1}=\left(\begin{array}{c} 1/2\\ -\sqrt{3}/2 \end{array}\right),\;R_{AC,2}=\left(\begin{array}{c} -1\\ 0 \end{array}\right),\;R_{AC,3}=\left(\begin{array}{c} 1/2\\ \sqrt{3}/2 \end{array}\right)\right)$ is rotated matrix; $f_{len}$ is the distance function of the pixel parameter $P_{img,i}$ and the center position in the horizontal direction:

(20) flen(Pimg)=norm(fdep(CV)⋅tan(Pimg,xkimg,X),fdep(CV)⋅tan(Pimg,ykimg,Y))

Then according to the following geometric relationship, the position of the contact point C*_i_* can be obtained:

(21) $$\begin{array}{l} nrom\left(l_{dep,Ci}-A_{img}\right)=\sqrt{l_{AC}^{2}+d_{C}^{2}}\\ A_{img}=\left(\begin{array}{c} r_{A}\\ f_{dep}\left(C_{R}\right)-h_{O} \end{array}\right)\\ h_{Ci}=f_{dep}(P_{img,i})-f_{dep}\left(C_{R}\right)+h_{O} \end{array}$$

where $A_{img}$ represents the coordinate position of point A*_i_* in the depth image; $h_{O}$ is the height of the robot center from the ground after landing.

#### 4.2.3. Driving Variable Analysis

When substituting the centrifugal height $h_{Ci}$ of the touch point (which is calculated by the touch point position) into the robot inverse kinematics (analyzed above), the joint variables of each driving joint can be solved:

(22) $$l_{Si}=F_{inverse}\left(h_{Ci}\right)$$

Finally, the calculated driving joint variables are input to the driving board. The driving board uses the displacement of the slider as the target value to perform PID control on the slider motor to realize the motion control of the robot.

## 5. Experimental Test

To verify the feasibility of the algorithm proposed in this article, this section will introduce the construction of the experimental platform, the experimental process, and the analysis of the experimental results in detail.

### 5.1. Experiment Platform and Process

**Figure 9:**
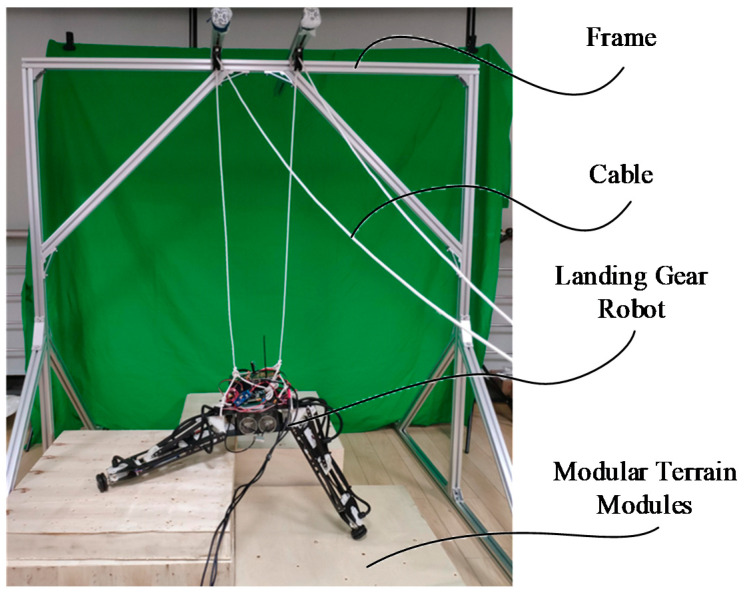
Experiment platform.

In this study, the verification experiment was mainly carried out on an indoor experimental platform. The experimental platform is shown in Figure 9: It is mainly composed of three parts—the frame, the cable, and the terrain platform. One end of the cable is fixed on the robot, and the other end is a free end that passes through the fixed pulley on the main bracket. By controlling the expansion and contraction of the cable, the robot’s landing process is simulated. The terrain platform is composed of modular terrain modules, which are combined with modules of different heights to form different terrains.

**Figure 10:**
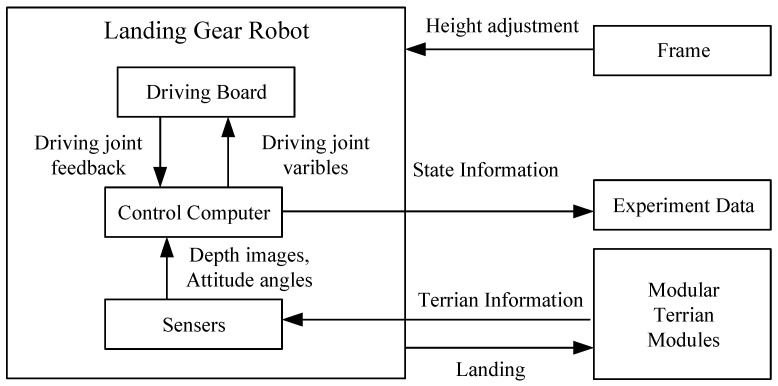
Flow chart of experiment data collection.

In this study, the robot’s take-off and landing process was simulated by releasing and pulling back cables. The entire adaptive landing process was monitored in real-time. The experimental process is shown in Figure 10.

When the robot is at the standby altitude, the sensor collects terrain depth information and the status information of the robot and transmits the information to the control computer for processing. The control computer calculates the driving joint variables of the robot according to the algorithm designed above, and outputs them to the driving board of the robot. The driving board executes the motion of the motors according to the driving joint variables, and feeds back the position parameters of the driving motors to the control computer in real-time.

The height of the robot is lowered by slowly releasing the cable, until the robot landed on the ground. The control computer will collect and record the robot attitude information, expected driving joint variable, actual driving joint variable, and center height of the entire experiment. It is used to analyze the adaptive landing function of robots.

In order to test the influence of terrain structure, ambient light and terrain color on the designed algorithm, three groups of contrast experiments were designed. The first group of experiments are built in a normal environment, but with different terrain structures. The second group is designed with different terrain colors. The last group is set with different ambient lights.

### 5.2. Results and Discussion

**Figure 11:**
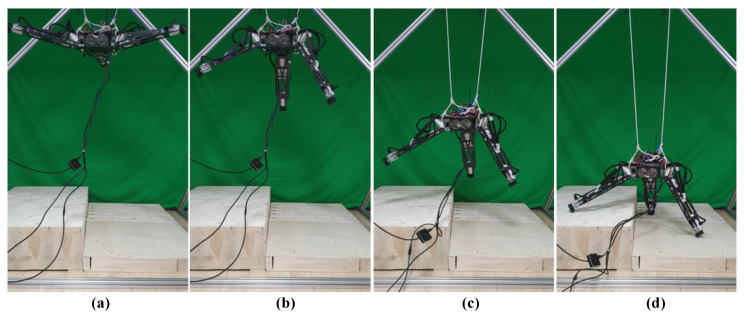
Adaptive landing process: (**a**) Initialization, (**b**) adjustment, (**c**) descend, (**d**) completeness.

This section presents the results and analysis of this experiment. Figure 11 shows the four process moments of the experiment: Initialization, adjustment, descend, and completeness.

**Figure 12:**
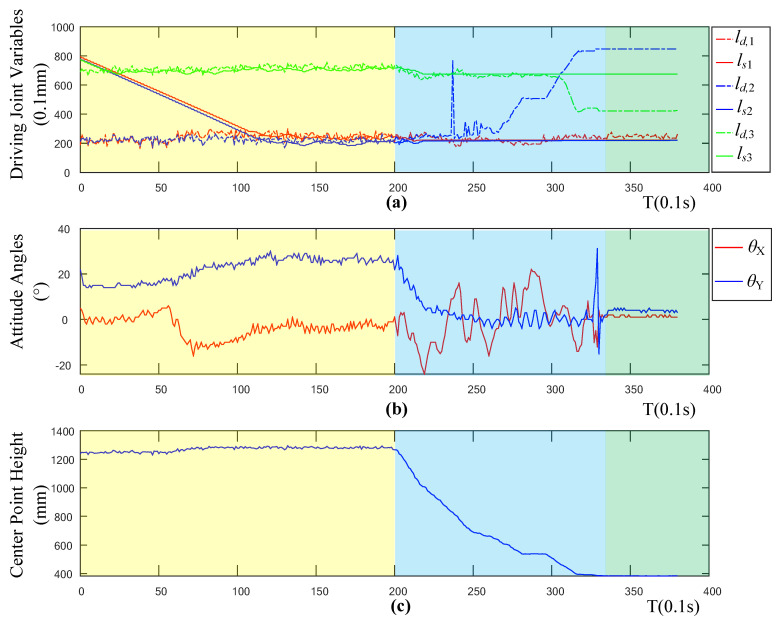
(**a**) Diagram of driving joint variables, (**b**) attitude angles, and (**c**) center point height.

Figure 12 shows the robot state variables collected during the entire experiment. The sub-figures in Figure 12 record the relationship between the driving joint variables, the attitude angle, and the height of the robot center position. In Figure 12c, according to the height curve of the robot, the landing process of the robot can be divided into three stages. The first stage (0–20 s, yellow region) is the landing preparation stage, in this stage, the drone will maintain a fixed altitude and fixed-point flight. The second stage (20–33 s, blue region) is the descent stage, in this stage, the drone lands vertically at a low speed. The third stage (33–40 s, green region) is the landing stabilization stage. At this time, the robot system has completed the entire landing process while keeping the height and attitude of the robot stable.

Figure 12b records the changes of the robot’s pitch $\theta_{Y}$ and roll angle $\theta_{X}$ during the descending phase. Corresponding to the three stages of the robot’s landing process, the angle of the robot changes greatly during the descending phase, due to the disturbance caused by the manual release of the cable. This is similar to the disturbance phenomenon when the drone is performing position control and attitude control during the landing at a fixed point. The robot has a small angle fluctuation during the preparation phase as the robot is influenced by a reaction force generated when the robot rotates its robotic arms.

Figure 12a shows the relationship between the expected driving joint variables *l_d,i_* (*i* = 1, 2, 3) and the actual joint variables *l_s,i_* throughout the landing process. The dotted line represents the driving joint variable curve calculated by the control computer through the control algorithm, and the solid line represents the driving joint variable fed back by the robot in real-time. In the preparation stage, the robot’s driving joint gradually approaches the expected position from the initial position, and finally reaches the curve of the expected position. In the descending phase, when the height is lower than 60 cm, the value of the drive variable calculated by the control computer begins to change significantly. This is because when the robot is landed to a certain height, the viewing angle of the depth camera is limited, and the landing point of the robot exceeds the calculation range of the viewing angle, thereby causing a disturbance. Therefore, in this study, the height of 100 cm is set as the threshold. When the height of the robot is higher than the threshold, the calculated drive value of the control computer is believed to be reliable, and the robot follows the calculated value. When the height is lower than the threshold, the robot maintains the last trusted value. The resulting curve trend is shown in Figure 12a. When the height is less than 100 cm, the actual joint variables no longer follow the expected joint variable movement.

**Figure 13:**
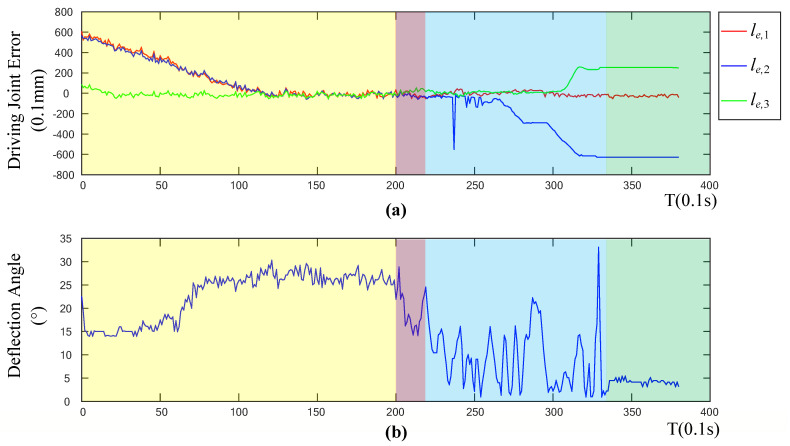
(**a**) Diagram of driving joint error, and (**b**) deflection angle.

To further explore the effectiveness of the adaptive landing algorithm, this study calculated the error curve of the driving joint variable and the curve of the robot’s tilt angle. It can be seen from Figure 13a that the variation trend of the error curve of the driving joint variables is basically consistent with the variation trend of the drive variable fed back by the robot. In the preparation stage, when the system is stable, there is zero error associated with the three driving joint variables, which shows the effectiveness of the robot motor driving algorithm. Figure 13b shows the deflection angle $\theta_{err}$ between the *Z* axis of the robot’s coordinate axis and the *Z* axis of the geodetic coordinate system. The calculation formula is as follows:

(23) $$\theta_{err}=\arccos\left(\cos\left(\theta_{X}\right)\cdot \cos\left(\theta_{Y}\right)\right)$$

It can be seen from Figure 13 that during the landing stabilization phase, the tilt angle of the robot is less than five degrees, and the landing can be considered as stable and horizontal. In the preparation phase and the landing phase, the tilt angle of the robot is larger, while in the final stabilized phase, the tilt angle is smaller. This proves that the adaptive control algorithm is less affected by the flight attitude change and speed change, which verifies the effectiveness of the control algorithm.

The changing curve of the robot’s posture angle in Figure 12 shows that the posture angle will vibrate with the movement of the robot. However, when the robot nearly reaches the expected position, it will still move in small amplitude with the change of the expected driving joint variable, and this high-frequency, small-amplitude vibration motion will have a greater impact on the flight stability of the flying robot.

**Figure 14:**
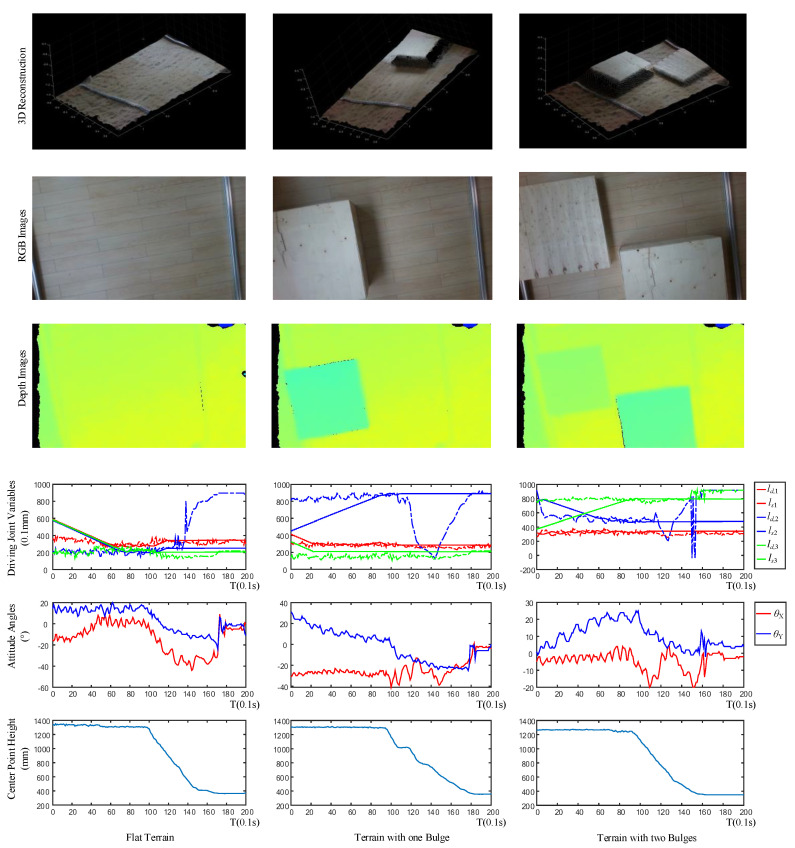
Experiments in different terrains.

Figure 14 demonstrates the results of the experiments in different terrains. The sub-figures in the first row shows the 3D reconstructions in 1-m height. The second and third row present the RGB images and the depth images separately. The curves of the driving joint variables, attitude angles, and center point height are shown in fourth, fifth, and sixth rows. Each column represents the corresponding experiments. In the first experiment, the terrain is flat, and all three driving joint variables are almost the same, which satisfies the flat terrain. In the second experiment, a bulge is set on the ground. One driving joint variable is bigger than the others to satisfy the terrain with one bulge. In the last experiment, one high bugle and one low bugle are set on the ground. All three driving joint variables are different owing to the terrain. These results show that the designed control systems can work well. On the other hand, when the robot landed, the attitude angle errors were small and between plus and minus 10 degrees, also shows the effectiveness of the designed system. The testing results show that the designed system work well in different rigid terrains.

**Figure 15:**
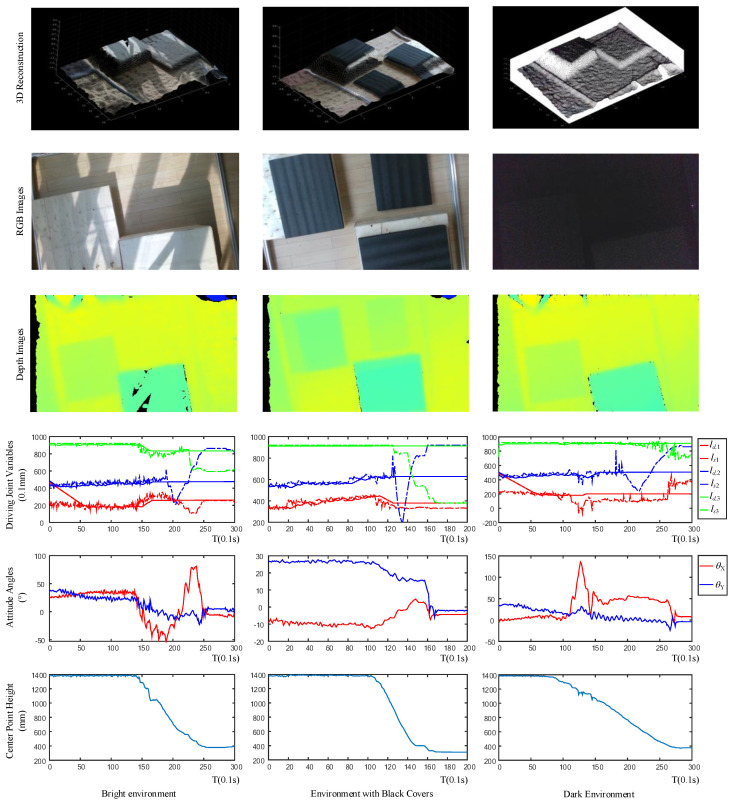
Experiments in different environmental conditions.

Figure 15 shows the results of the experiments in different environmental conditions. Just like Figure 14, the sub-figures in different rows also show the results of 3D reconstructions, RGB images, depth images, driving joint variables, attitude angles, and center point height separately, while each column represents the corresponding experiments in different environmental conditions. In the first column, the figures present the results of the experiment with bright sunlight. The bright sunlight and shadow are clear in the RGB image, and the bright sunlight does influence the depth camera, which causes noise in the 3D reconstruction and the depth image. However, the attitude angle curves show that the angle error are still small, which means the effect of bright sunlight is restricted. In the second experiment, the terrain is covered by black foams. However, the change of terrain color has no impact on the depth camera nor the control system. In the last column, the experiment is tested in the dark environment. The terrain cannot be distinguished from the RGB image, but are still clear in the 3D reconstruction and the depth image. Moreover, the attitude angle curves also show darkness has no impact on the control system. All the results show that the ambient light and the terrain color have a limited impact on the designed control system.

**Figure 16:**
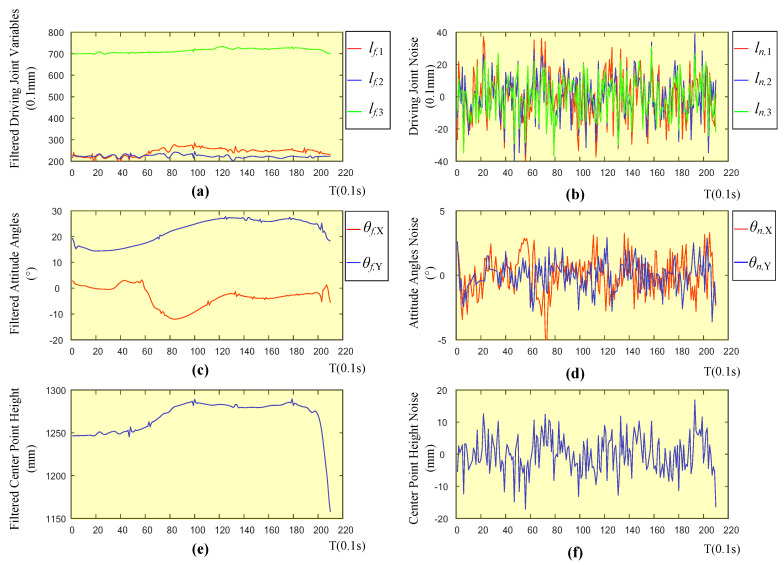
(**a**) The filtered curve of driving joint variables, (**c**) attitude angles, (**e**) center point height, and (**b**) noise curve of driving joint variables, (**d**) attitude angles, (**f**) center point height.

In order to determine the specific cause of the expected joint jitter, this study continues to analyze the relationship between the expected driving joint variable and the depth information and attitude angle. In this study, the expected driving joint variables, depth of the center point, and attitude angle curves are regarded as three kinds of signals, and the jitter of the signals is caused by noise. Therefore, in this article, the noise signals of the three signals are separated by wavelet filtering, and a comparative analysis is performed to determine the source of the expected driving joint variable noise. Figure 16 shows the filtered signal and noise signal of the driving joint signal, center position height signal, and inclination signal during the preparation stage. It can be seen from the figure that the filtered signal retains the movement trend of the original signal, but the change is relatively smooth. To analyze the correlation between various noise signals, this paper analyzes the Pearson correlation coefficient [24] among the three signals.

(24) $$\rho M,N=\frac{\mathrm{cov}(M,N)}{\sigma M\sigma N}=\frac{E\left(MN\right)-E\left(M\right)E\left(N\right)}{\sqrt{E\left(M^{2}\right)-E^{2}\left(M\right)}\sqrt{E\left(N^{2}\right)-E^{2}\left(N\right)}}$$

where cov(M,N) represents the covariance of the signal $M,N=h_{nO},l_{n,1},l_{n,2},l_{n,3},\theta_{n,X},\theta_{n,Y}$, and σ represents the standard deviation of the signal.

As shown in Table 2, it can be seen from the results that the driving joint variable is highly correlated with the noise signal at the center position height, which are, 0.7871, 0.7602, and 0.8064, respectively, but the noise signal correlation with the corner signal is relatively small. The height data of the center position comes from the depth sensor, so the main cause for the vibration of the driving variable is the signal noise of the depth sensor. By optimizing the depth solution algorithm of the depth sensor or filtering the depth image, the vibration of the driving joint can be alleviated.

From the experiments, the results verify that the designed control algorithm can achieve adaptive landing in different rigid terrains, and the ambient light and the terrain color have a limited impact on the designed control system. However, some problems are also explored in the experiments. First, noise signals from the depth camera cause an error in robot’s kinematical analysis. Second, visual angles of the depth camera limit the control algorithm when vehicles go down below the threshold height. Third, ambient light causes the noise in the depth camera. We will keep working on solving these three problems and testing the system on more terrains in the future.

## 6. Conclusions

In order to expand the take-off and landing functions of uncrewed aerial vehicles in a non-structural responsible environment, this study designs an adaptive landing control system for our self-developed robots. The control system uses multi-sensor fusion technology to realize three-dimensional terrain scanning of the landing area. To accurately calculate the robot’s landing position, the control system uses a Kalman filter and complementary filter algorithms to fuse the robot’s inclination and speed information. Then, based on the robot kinematics model, the robot’s landing posture and driving joint variables are determined. Finally, this article verifies the feasibility and effectiveness of the adaptive landing control system through experiments. Based on the analysis of experimental data, the causes of the robot’s lower abdominal concussion are discussed, which provides a basis for further optimization of the robot control algorithm design.

## Figures and Tables

**Table 1:** Dimension parameters of the landing gear robot.

Parameter	Value	Parameter	Value
*r*_A_	170 mm	*l*_AC_	415 mm
*r*_E_	220 mm	*d*_C_	40 mm
*h*_AE_	65 mm	*l*_DE_	195 mm
*d*_S_	22 mm		

**Table 2:** Pearson correlation coefficient.

Coefficient	$l_{n,1}$	$l_{n,2}$	$l_{n,3}$
$h_{nO}$	0.7871	0.7602	0.8064
$\theta_{n,X}$	−0.1398	−0.0615	0.0247
$\theta_{n,Y}$	0.1228	0.0421	0.1124
